# Depletion of the Antioxidant Ergothioneine by Critical Illness and Continuous Renal Replacement Therapy

**DOI:** 10.3390/kidneydial6030048

**Published:** 2026-07-14

**Authors:** William A. Vasquez Espinosa, Seolhyun Lee, Josef K. Suba, Lindsey S. Keo, Timothy W. Meyer, Tammy L. Sirich

**Affiliations:** 1 Department of Medicine, Stanford University, Palo Alto, CA 94304, USA; 2 Department of Medicine, Veterans Affairs Palo Alto Healthcare System, Palo Alto, CA 94304, USA

**Keywords:** antioxidant, continuous renal replacement therapy, nutrition

## Abstract

**Background:**

Ergothioneine is a diet-derived antioxidant with a specific membrane transporter that uptakes it into tissues susceptible to oxidative stress. We previously found that efficient dialytic clearance leads to marked ergothioneine depletion in patients on hemodialysis. Continuous renal replacement therapy (CRRT) provides higher time-averaged clearances of small solutes than hemodialysis. We therefore examined whether ergothioneine is depleted in patients receiving CRRT.

**Methods:**

We first compared erythrocyte and plasma ergothioneine levels in 19 critically ill subjects maintained on CRRT for ≥2 days (CRRT), 18 critically ill subjects without kidney impairment (CI), and 15 healthy subjects (control). We then examined the degree to which initiation of CRRT reduced ergothioneine levels in a separate group of 11 subjects after ≥2 days on CRRT and measured ergothioneine in the effluent fluid to calculate its CRRT clearance.

**Results:**

Compared to controls, erythrocyte and plasma ergothioneine levels were depleted in both critically ill subjects maintained on CRRT for 20 ± 26 days and critically ill subjects not on CRRT (erythrocyte: CRRT 226 ± 121 μM, CI 208 ± 132 μM, control 593 ± 568 μM; plasma: CRRT 0.52 ± 0.37 μM, CI 0.86 ± 0.83 μM, control 2.3 ± 1.7 μM). Initiation of CRRT in a separate group of 11 subjects significantly reduced the plasma level but not the erythrocyte level of ergothioneine after 3.4 ± 1.0 days. CRRT provided similar efficient clearances of ergothioneine, urea, and creatinine (31 ± 8, 38 ± 8, and 36 ± 10 mL/min).

**Conclusions:**

Ergothioneine is significantly depleted in critically illness, and prolonged CRRT may worsen the depletion. Our findings motivate further investigation of the impact of ergothioneine depletion in critical illness.

## 1. Introduction

Critically ill patients often require continuous renal replacement therapy (CRRT) to manage electrolyte and fluid status [1,2]. CRRT also removes potentially harmful waste solutes that accumulate when the kidney function is impaired. However, CRRT removes solutes indiscriminately, risking the loss of valuable solutes. Indeed, previous studies have shown that CRRT removes a substantial amount of small amino acids, trace elements, and vitamins, and a recent study showed that CRRT also removes thyroid stimulating hormone and free thyroxine [3–9]. Patients who require CRRT have increased mortality and morbidity [1,10]. While these poor outcomes are largely due to the severity of the underlying critical illness, the depletion of valuable solutes by CRRT could contribute.

In recent efforts to identify solutes depleted by renal replacement therapies, we found that ergothioneine was markedly deficient in patients with end-stage kidney disease receiving hemodialysis and peritoneal dialysis [11,12]. While dialysis can deplete many potentially valuable solutes, we focused on ergothioneine given the accumulating evidence of its biologic value. Ergothioneine is a diet-derived antioxidant with a highly specific membrane transporter that uptakes it into tissues susceptible to oxidative stress [13]. Indeed, erythrocytes have high concentrations of ergothioneine at approximately 200-fold that of its plasma concentrations [11,12,14]. Erythrocytes are constantly exposed to reactive oxygen species and radicals produced by the interaction of hemoglobin with oxygen [15]. In vitro and in vivo studies have demonstrated ergothioneine can reduce oxidative and inflammatory injury [13,16]. Lower ergothioneine levels have been associated with neurodegenerative and cardiovascular diseases [13]. Furthermore, recent trials have shown that ergothioneine supplementation improves sleep and cognition in people without kidney disease [17].

Both hemodialysis and peritoneal dialysis indiscriminately remove ergothioneine, a small unbound solute with a molecular mass of 229 Da and an effective volume of distribution similar to that of creatinine [11]. We found that this indiscriminate removal accounts for ergothioneine depletion in patients maintained on dialysis [11,12]. The efficiency of solute clearance by CRRT depends on the modality (hemofiltration, dialysis, or a combination of both as hemodiafiltration) and characteristics of the solute [18]. Clearance by hemofiltration is determined by the rate of ultrafiltration, and solutes get dragged along with the fluid down a pressure gradient via convective clearance. Clearance by dialysis is determined by the rate of dialysate flow, and solutes move down a solute concentration gradient via diffusive clearance. Small solutes (molecular mass < 500 Da) that are not bound to plasma proteins are cleared with similar efficiency by convection and diffusion [6,19,20]. At a given CRRT dose, different CRRT modalities would therefore provide approximately the same clearances of small solutes including ergothioneine, an unbound solute with molecular mass of 229 Da. The currently recommended CRRT dose of 20–25 mL/kg/hour provides a much higher time-averaged clearance for small solutes like ergothioneine than conventional hemodialysis and peritoneal dialysis [2]. In this study, we therefore examined whether ergothioneine is depleted in patients receiving CRRT.

## 2. Materials and Methods

### 2.1. Recruitment and Sample Collection

The first aim of our study was to examine the degree of ergothioneine depletion in patients maintained on CRRT. We compared three groups of subjects: 19 critically ill subjects maintained on CRRT (CRRT), 18 critically ill subjects without kidney impairment (CI), and 15 healthy subjects with no known kidney disease (control). The CRRT and CI subjects were recruited at Stanford Hospital between August 2024 and May 2025. Patients were eligible if they were ≥18 years old, were not receiving chronic dialysis prior to hospital admission, were not participating in another study, and were deemed suitable candidates for a research study by their primary providers. An additional criterion for CRRT subjects was maintenance on CRRT for at least 2 days. The CRRT dose of 26 ± 3 mL/kg/hour was provided in these 19 subjects as continuous venovenous hemodiafiltration (25 ± 14% pre-filter replacement fluid, 25 ± 12% post-filter replacement fluid, and 50 ± 19% dialysate fluid). A standard protocol was employed for citrate anticoagulation. An additional criterion for CI subjects was no evidence of kidney impairment, defined as an increase in plasma creatinine > 0.3 mg/dL within the prior 48 h, a 1.5-fold increase in plasma creatinine within the prior 7 days, or a plasma creatinine of >1.3 mg/dL. Control subjects were from our prior studies and had been recruited if they reported no known kidney disease [12]. All subjects (CRRT, CI, and control) provided one blood sample.

The further aim of our study was to examine whether initiation of CRRT reduced plasma and/or erythrocyte ergothioneine levels. We recruited a separate group of subjects for whom CRRT initiation was planned. These subjects were recruited at Stanford Hospital during the same period as the CRRT and CI subjects. Subjects were eligible if they were ≥18 years old, were not participating in another study, and were deemed suitable candidates for a research study by their primary providers. Blood collection was obtained at CRRT initiation (baseline) and again at least 2 days after CRRT initiation (repeat). CRRT effluent fluid was collected at the same time as the repeat blood collection. Sixteen subjects were recruited in this group, of whom 11 were included and 5 were excluded. Reasons for exclusion of the 5 subjects were as follows: 1 was prescribed an atypically high CRRT dose (84 mL/kg/hour) for liver failure, 2 expired before the repeat blood sample could be obtained, and 2 were taken off CRRT before the repeat blood sample could be obtained. The CRRT dose averaging 25 ± 1 mL/kg/hour was provided in these 11 subjects as continuous venovenous hemodiafiltration (28 ± 6% pre-filter replacement fluid, 31 ± 11% post-filter replacement fluid, and 40 ± 14% dialysate fluid).

The study was approved by the institutional review board of Stanford University and was conducted in accordance with the Declaration of Helsinki. Informed consent was obtained from all participants or their legally authorized representative if applicable.

### 2.2. Solute Measurements and Calculations

Aliquots of whole blood, plasma, and CRRT effluent were stored at −80 °C until analysis. Ergothioneine levels for all samples were measured using liquid chromatography tandem mass spectrometry with stable isotope dilution as previously described [11]. Standard curves were constructed to quantify ergothioneine levels, and quality control samples were analyzed with every sample run. We measured ergothioneine levels in both the whole blood and plasma. Whole blood levels allowed for the calculation of ergothioneine levels in the erythrocytes where ergothioneine is highly concentrated [11]. Plasma and CRRT effluent levels of urea nitrogen and creatinine were measured by the clinical laboratory.

CRRT clearance was calculated as the removal rate divided by the plasma level of each solute (ergothioneine, urea, creatinine). The removal rate was calculated as the effluent solute concentration multiplied by the total effluent flow rate (pre- and post-filter replacement fluid, dialysate fluid, and net ultrafiltration rates). The effluent-to-plasma ratio of each solute was calculated as the CRRT effluent concentration divided by the plasma concentration.

### 2.3. Statistical Analysis

The normality of data was assessed using the Shapiro–Wilks test, and log transformation was performed for data that were not normally distributed. Comparisons among the CRRT, CI, and control groups were performed using ANOVA, with significance of pairwise comparisons using the Tukey method and adjusted for multiple comparisons using the Bonferroni method. Comparisons between the CRRT and CI groups were performed using the unpaired *t*-test. The effect of CRRT on baseline versus repeat solute levels was compared using the paired *t*-test. Comparisons of CRRT clearance and effluent-to-plasma ratios among the solutes, ergothioneine, urea nitrogen, and creatinine, were compared using repeated measures ANOVA and adjusted for multiple comparisons using the Bonferroni method. Association of solute levels with days on CRRT and with days in the intensive care unit was performed using the Spearman’s Rank Correlation test. Statistical analysis was performed using SPSS v31 (Chicago, IL, USA).

## 3. Results

### 3.1. Demographics and Clinical Data of CRRT, CI, and Control Subjects

Table 1 summarizes the demographic and clinical data for the CRRT, CI, and control subjects. The age, sex, weight, and body surface area were similar among the three groups. The duration of overall hospitalization, represented as from the day of hospital admission to the day of the study sample collection, varied widely but was not different between the CRRT and CI groups. The CRRT subjects, however, had more intensive care unit days (47 ± 66 vs. 9 ± 15 days) with provision of CRRT for 20 ± 26 days (range 2 to 110 days). The CRRT subjects also had higher Sequential Organ Failure Assessment (SOFA) scores than the CI subjects (13.3 ± 2.7 vs. 5.7 ± 2.5). The SOFA score of the CRRT subjects remained higher even after excluding the kidney subscore (9.3 ± 2.7 vs. 5.7 ± 2.5). Provision of CRRT kept the plasma levels of potassium, HCO_3_, and phosphorus similar to the CI group, as summarized in Supplementary Table S1. Plasma albumin and hemoglobin levels were also similar. Reasons for hospitalization for the CRRT and CI subjects are summarized in the Supplementary Material.

### 3.2. Solute Levels

**Figure 1.**
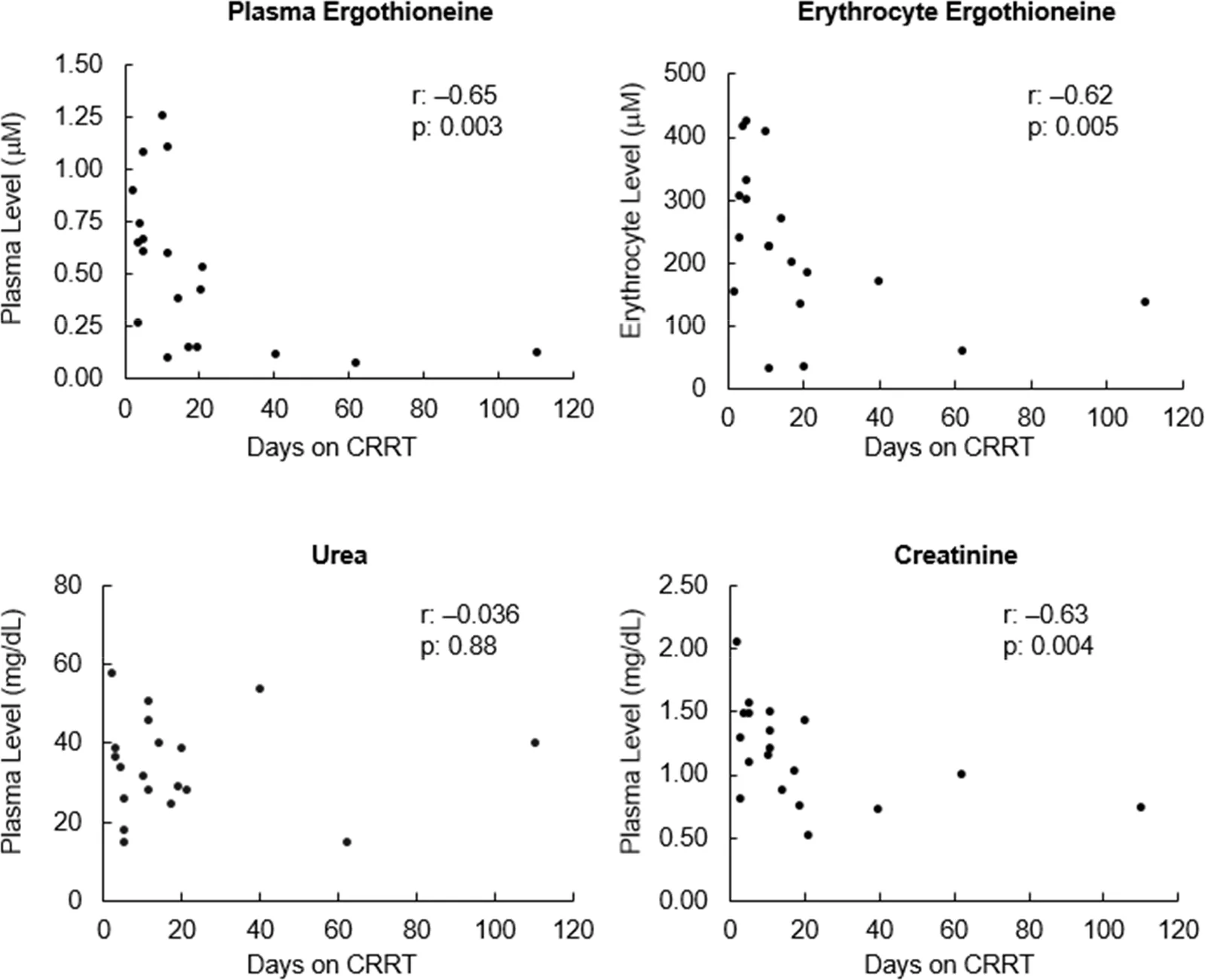
The relationship between levels of ergothioneine (erythrocyte and plasma), urea, and creatinine with days on CRRT is shown for the 19 subjects who were maintained on CRRT for at least 2 days. Each dot represents a CRRT subject.

Table 2 summarizes the urea nitrogen, creatinine, and ergothioneine levels in the CRRT, CI, and control subjects. The plasma urea nitrogen levels of the CRRT subjects were higher than in control subjects but not different from in the CI subjects. Provision of CRRT maintained the plasma creatinine levels of the CRRT subjects similar to the controls. In contrast, the plasma ergothioneine levels in both the CRRT and CI subjects were lower than in the control subjects (CRRT: 0.52 ± 0.37 μM, CI: 0.86 ± 0.83 μM, control: 2.3 ± 1.7 μM). Like the plasma levels, erythrocyte ergothioneine levels in both the CRRT and CI subjects were lower than in the control subjects (CRRT: 226 ± 121 μM, CI: 208 ± 132 μM, control: 593 ± 568 μM). The average plasma and erythrocyte ergothioneine levels in CRRT subjects were not lower than in CI subjects. However, a longer duration of CRRT was associated with lower levels of both plasma ergothioneine (r: −0.65, *p* 0.003) and erythrocyte ergothioneine (r: −0.62, *p* 0.005), as shown in Figure 1. The association persisted after excluding two subjects with the longest CRRT duration of 62 and 110 days (plasma ergothioneine: r −0.54, *p* 0.025; erythrocyte ergothioneine: r −0.54, *p* 0.027). Prolonged CRRT was also associated with lower plasma creatinine levels but not with lower plasma urea nitrogen levels, as further shown in Figure 1. Plasma and erythrocyte ergothioneine levels were not associated with the duration of intensive care in the CI subjects (Supplementary Figure S1).

### 3.3. Effect of CRRT Initiation

The effect of CRRT initiation on the plasma levels of urea nitrogen and creatinine and on the plasma and erythrocyte levels of ergothioneine in the separate group of 11 subjects is summarized in Table 3. Demographic data and laboratory values for these subjects are summarized in Supplementary Table S2. Baseline blood was collected 1.8 ± 2.6 h before initiating CRRT for 10 patients and 0.5 h after initiating CRRT for 1 patient. A repeat blood sample and simultaneous CRRT effluent were collected 3.4 ± 1.0 days (range 2 to 5 days) after initiating CRRT. Initiation of CRRT reduced the plasma levels of urea nitrogen and creatinine after 3.4 ± 1.0 days. CRRT also reduced the plasma levels of ergothioneine, but not the erythrocyte levels of ergothioneine.

Table 4 summarizes the clearance by CRRT and the effluent/plasma ratios for urea, creatinine, and ergothioneine. CRRT provided similar clearances for all three solutes (urea: 38 ± 8 mL/min; creatinine: 36 ± 10 mL/min; ergothioneine: 31 ± 8 mL/min). The effluent/plasma ratios were also similar among the three solutes (urea: 1.02 ± 0.16; creatinine: 0.97 ± 0.21; ergothioneine: 0.86 ± 0.20). The effluent concentrations of the solutes are summarized in Supplementary Table S3.

## 4. Discussion

Kidney impairment requiring CRRT is a common consequence of critical illness that carries high morbidity and mortality [1,10]. While reasons for poor outcomes of patients requiring CRRT are undoubtedly complex and dominated by the underlying critical illness, the indiscriminate removal and subsequent depletion of valuable solutes by CRRT may contribute. We previously reported significant ergothioneine depletion in patients maintained on hemodialysis and peritoneal dialysis [11,12]. While many solutes are at risk of depletion by renal replacement therapy, we focused our efforts on ergothioneine because of its potential biologic value. It is an antioxidant that is taken up in high concentrations into tissues that are vulnerable to oxidative injury such as erythrocytes, mitochondria, and brain [13,21]. Ergothioneine is made primarily by fungi, and humans therefore obtain it exclusively from their diet. Strong support of its importance is the existence of the membrane transporter SLC22A4 that is highly specific for ergothioneine [22]. Indeed, SLC22A4’s affinity for ergothioneine is several-fold higher than numerous other tested compounds. This transporter is abundant in the intestines to absorb ergothioneine from the diet [13,22]. It is present in the kidney tubules and avidly reabsorbs ergothioneine so that very little is lost in the urine [11,14]. It is also expressed abundantly in precursor erythrocytes and takes up ergothioneine before differentiation into mature erythrocytes [15,22]. As demonstrated in knockout mice, this transporter is required for ergothioneine uptake from the plasma into various tissues [22]. In addition, the clearance of ergothioneine by hemodialysis is similar to that of creatinine and lower than urea, indicating that ergothioneine does not diffuse out of erythrocytes during blood passage through the dialyzer membrane [11].

In this exploratory study, we found that ergothioneine levels were markedly low in the erythrocytes and plasma of subjects maintained on CRRT. We were intrigued to find that ergothioneine levels were also low in the critically ill subjects who were not receiving CRRT. Indeed, decreased levels of other antioxidants such as glutathione have been observed in critical illness [23]. We hypothesize that some combination of reduced intake and of loss not occasioned by CRRT causes ergothioneine depletion in critical illness. Because humans cannot make ergothioneine, its levels are dependent on intake of foods that contain ergothioneine. Intake of ergothioneine may be reduced during critical illness, as patients may require specific diets and/or enteral supplementation that do not contain ergothioneine. The intestinal absorption of ergothioneine could also be impaired in critical illness. In mice, lower ergothioneine levels in chronic kidney disease have been attributed to impaired absorption from reduced expression of the SLC22A4 transporter on intestinal apical cell membranes [24]. Given ergothioneine’s antioxidant effects, its uptake into various tissues may be increased in the face of acute oxidative stress that develops with critical illness [13,25–27]. Indeed, in mice, SLC22A4 transporter expression was increased in response to tissue injury, thereby increasing ergothioneine uptake into the damaged tissue [13,24,28]. A recent study also showed that ergothioneine accumulated in muscle mitochondria of mice during exercise training [29]. Ongoing oxidative stress during critical illness could increase the preferential tissue uptake of ergothioneine [27]. The CRRT subjects in this study had worse SOFA scores and longer intensive care unit stay than the CI subjects; the severity of illness could contribute to the depletion of ergothioneine independent of CRRT. The combination of increased demand for antioxidant effects, reduced dietary intake, and impaired intestinal absorption may therefore deplete ergothioneine reserves in various tissues.

Prolonged provision of CRRT could further worsen ergothioneine depletion in critical illness. We found that CRRT provided similar efficient clearance of ergothioneine as that of urea and creatinine, reducing the plasma levels of all three solutes within approximately 3 days of CRRT initiation. Of interest, however, the erythrocyte levels of ergothioneine did not decline after CRRT initiation. This may be explained by erythrocytes having received their entire complement of ergothioneine as precursor erythrocytes before their release from the bone marrow. Indeed, current data suggest that mature erythrocytes in the circulation do not have the SLC22A4 uptake transporter [15]. Erythrocyte ergothioneine levels may eventually decline with continued plasma depletion by CRRT, as less ergothioneine would be available in the plasma for uptake into new precursor erythrocytes. Ergothioneine levels may also decline in other tissues such as the brain and mitochondria when its plasma levels are reduced further by the initiation of CRRT. However, we did not measure ergothioneine in any tissues other than the erythrocyte and so could not test this. At current recommended CRRT doses, the time-averaged clearance of small solutes like ergothioneine provided by CRRT is much higher than that provided by conventional hemodialysis [11]. If lower doses of CRRT prove to be effective, loss of ergothioneine and other valuable solutes would be less, but depletion may still occur with prolonged CRRT [30–34]. While critically ill patients on CRRT are prescribed higher intakes of protein and micronutrients to offset CRRT losses, there are currently no data to support the inclusion of ergothioneine in nutritional supplements [3,6,26,35].

## 5. Limitations

Our study has limitations. It was a small single-center study and therefore cannot provide meaningful relationships between ergothioneine levels and clinical outcomes. We did not measure ergothioneine levels beyond five days in the subjects initiating CRRT and therefore cannot confirm the extent to which prolonged CRRT reduces plasma and erythrocyte levels of ergothioneine in individual subjects. Sixteen CRRT subjects and seven CI subjects received packed red blood cell transfusions prior to study sample collection, and five subjects who initiated CRRT received packed red blood cell transfusions between their baseline and repeat blood collections. Transfused red cells may contain ergothioneine and therefore affect patients’ average erythrocyte levels; the content of ergothioneine in stored erythrocytes and whether ergothioneine degrades over time in stored erythrocytes, however, have not been assessed [15]. Ergothioneine levels in the healthy control group were from our previous studies and were not collected during the same recruitment period as the CRRT and CI subjects. Finally, we did not obtain detailed dietary data from the critically ill subjects prior to their hospitalization.

## 6. Conclusions

In conclusion, the antioxidant ergothioneine is markedly depleted in critically ill patients regardless of whether or not they were receiving CRRT. However, prolonged provision of CRRT may risk further loss of ergothioneine. Given ergothioneine’s antioxidant effects and its potential biologic value, our preliminary findings motivate further investigation of the clinical impact of ergothioneine depletion in critical illness.

## Supplementary Material

Supplementary Material

**Supplementary Materials:** The following supporting information can be downloaded at: https://www.mdpi.com/article/10.3390/kidneydial6030048/s1, Supplementary Materials: Reasons for hospitalization of the CRRT Subjects, CI Subjects, and Subjects Initiating CRRT; Supplementary Table S1: Laboratory Data for CRRT and CI Subjects; Supplementary Table S2: Demographics and Laboratory Values for 11 Subjects Initiating CRRT; Supplementary Table S3: CRRT Effluent Levels of Solutes for 11 Subjects Initiating CRRT; Supplementary Figure S1: Association of Ergothioneine Levels with Length of Intensive Care Unit Stay in Critically Ill Subjects.

## Figures and Tables

**Table 1.** Demographics and clinical data.

	CRRT (n = 19)	CI (n = 18)	Control (n = 15)
Age (years)	54 ± 17	62 ± 14	59 ± 12
Sex (M/F)	12/7	11/7	11/4
Weight (kg)	71 ± 16	78 ± 21	77 ± 14
Body surface area (m^2^)	1.64 ± 0.42	1.87 ± 0.24	1.92 ± 0.19
Days of hospitalization (no.)	49 ± 66	28 ± 56	-
Days in intensive care unit (no.)	47 ± 66 ^a^	9 ± 15	-
Days on CRRT (no.)	20 ± 26	-	-
SOFA score	13.3 ± 2.7 ^a^	5.7 ± 2.5	
SOFA score (excluding kidney subscore)	9.3 ± 2.7 ^a^	5.7 ± 2.5	-

a , *p* < 0.05 CRRT vs. CI. results are mean ± standard deviation. CRRT, continuous renal replacement therapy; CI, critically ill; SOFA, Sequential Organ Failure Assessment. The SOFA score was calculated on the day of study sample collection.

**Table 2.** Solute levels in CRRT, CI, and control subjects.

	CRRT (n = 19)	CI (n = 18)	Control (n = 15)
Plasma Urea Nitrogen (mg/dL)	34 ± 12 ^a^	29 ± 24	16 ± 4
Plasma Creatinine (mg/dL)	1.17 ± 0.38 ^b^	0.77 ± 0.19	0.92 ± 0.21
Plasma Ergothioneine (μM)	0.52 ± 0.37 ^a^	0.86 ± 0.83 ^c^	2.3 ± 1.7
Erythrocyte Ergothioneine (μM)	226 ± 121 ^a^	208 ± 132 ^c^	593 ± 568

a , *p* < 0.05 CRRT vs. control;

b , *p* < 0.05 CRRT vs. CI;

c , *p* < 0.05 CI vs. control. Results are mean ± standard deviation. CRRT, continuous renal replacement therapy; CI, critically ill.

**Table 3.** Effect of CRRT on solute levels.

Solute	Baseline	Repeat	Ratio of Repeat/Baseline
Plasma Urea Nitrogen (mg/dL)	57 ± 25 *	29 ± 11	0.54 ± 0.14
Plasma Creatinine (mg/dL)	4.2 ± 2.3 *	2.2 ± 0.8	0.63 ± 0.34
Plasma Ergothioneine (μM)	0.83 ± 1.0 *	0.54 ± 0.40	0.80 ± 0.26
Erythrocyte Ergothioneine (μM)	253 ± 131	232 ± 106	1.0 ± 0.43

* *p* < 0.05, baseline vs. repeat. Results are mean ± standard deviation. Data are shown for the 11 subjects who initiated CRRT.

**Table 4.** Solute clearances by CRRT.

	Urea	Creatinine	Ergothioneine
CRRT Clearance (mL/min)	38 ± 8	36 ± 10	31 ± 8
Effluent/Plasma Ratio	1.03 ± 0.16	0.97 ± 0.21	0.86 ± 0.20

Results are mean ± standard deviation. CRRT, continuous renal replacement therapy. Data are shown for 11 patients who initiated CRRT.

## Data Availability

Data will be made available at the time of publication to investigators who provide a methodologically sound proposal which has been approved by an independent review committee. Proposals should be submitted to tsirich@stanford.edu.
