# Supplementary material for: Depletion of the Antioxidant Ergothioneine by Critical Illness and Continuous Renal Replacement Therapy
Source: Kidney Dial. Author manuscript; Available in PMC 2026 Aug 27. (PMC13505502; doi:10.3390/kidneydial6030048)
Supplement: Supplementary file 1 [file NIHMS2195978-supplement-Supplementary_Material.pdf]

Supplementary Materials

Table of Contents

Content	Page
Reasons for hospitalization of the CRRT Subjects, CI Subjects, and Subjects Initiating CRRT	2
Supplementary Table 1 – Laboratory Data for 19 CRRT and 18 CI Subjects	3
Supplementary Table 2 – Demographics and Laboratory Values for 11 Subjects Initiating CRRT	4
Supplementary Table 3 – CRRT Effluent Levels of Solutes for 11 Subjects Initiating CRRT	5
Supplementary Figure 1 – Association of Ergothioneine Levels with Length of Intensive Care Unit Stay in Critically Ill Subjects	6

CRRT, continuous renal replacement therapy; CI: critically ill.

Reasons for hospitalization of the CRRT Subjects, CI Subjects, and Subjects Initiating CRRT

19 CRRT subjects:
Acute pancreatitis (1)
Cardiogenic compromise (6)
Endocarditis (2)
Hemorrhagic shock (1)
Interstitial lung disease; lung transplant (1)
Liver failure (4)
Pneumonia (2)
Urinary tract infection (2)

18 CI subjects:
B cell lymphoma (1)
Cardiogenic compromise (9)
Cholangitis (1)
Diverticulitis (1)
Kaposi sarcoma (1)
Liver failure (2)
Pneumonia (2)
Small bowel obstruction (1)

11 subjects initiating CRRT
Cardiogenic compromise (9)
Liver failure (2)

Supplementary Table S1 – Laboratory Data for CRRT and CI Subjects

**Laboratory Measure**	**CRRT (n=19)**	**CI (n=18)**
Potassium (mEq/L)	4.2 ± 0.7	4.2 ± 0.5
HCO_3 (mEq/L)	25 ± 4	25 ± 5
Phosphorus (mg/dL)	3.3 ± 0.8	3.3 ± 1.1
Albumin (g/dL)	3.1 ± 0.7	2.9 ± 0.5
Hemoglobin (g/dL)	9.0 ± 1.3	9.0 ± 1.4

Results are mean ± standard deviation. CRRT, continuous renal replacement therapy; CI: critically ill.

Supplementary Table S2 –

Demographics and Laboratory Values for 11 Subjects Initiating CRRT

**Characteristic**	**Value**
Age (years)	54 ± 17
Sex (no., M / F)	8 / 3
Body surface area (m^2)	2.12 ± 0.49
SOFA score	10.3 ± 4.1
Days of hospitalization (no.)	6.8 ± 4.9
Potassium (mEq/L)	4.4 ± 1.0
HCO_3 (mEq/L)	20 ± 6
Phosphorus (mg/dL)	5.0 ± 1.6
Albumin (g/dL)	3.3 ± 0.8
Hemoglobin (g/dL)	8.5 ± 1.7

Results are mean ± standard deviation. SOFA, Sequential Organ Failure Assessment. The SOFA score was calculated on the day of study sample collection.

Supplementary Table S3 –

CRRT Effluent Levels of Solutes for 11 Subjects Initiating CRRT

	**Urea (mg/dL)**	**Creatinine (mg/dL)**	**Ergothioneine (μM)**
CRRT Effluent Level	30 ± 12	2.0 ± 0.6	0.46 ± 0.37

Results are mean ± standard deviation. CRRT, continuous renal replacement therapy. The CRRT effluent fluid was collected at the time of the repeat blood sample collection.

Supplementary Figure S1 –

Association of Ergothioneine Levels with Length of Intensive Care Unit Stay

In Critically Ill Subjects

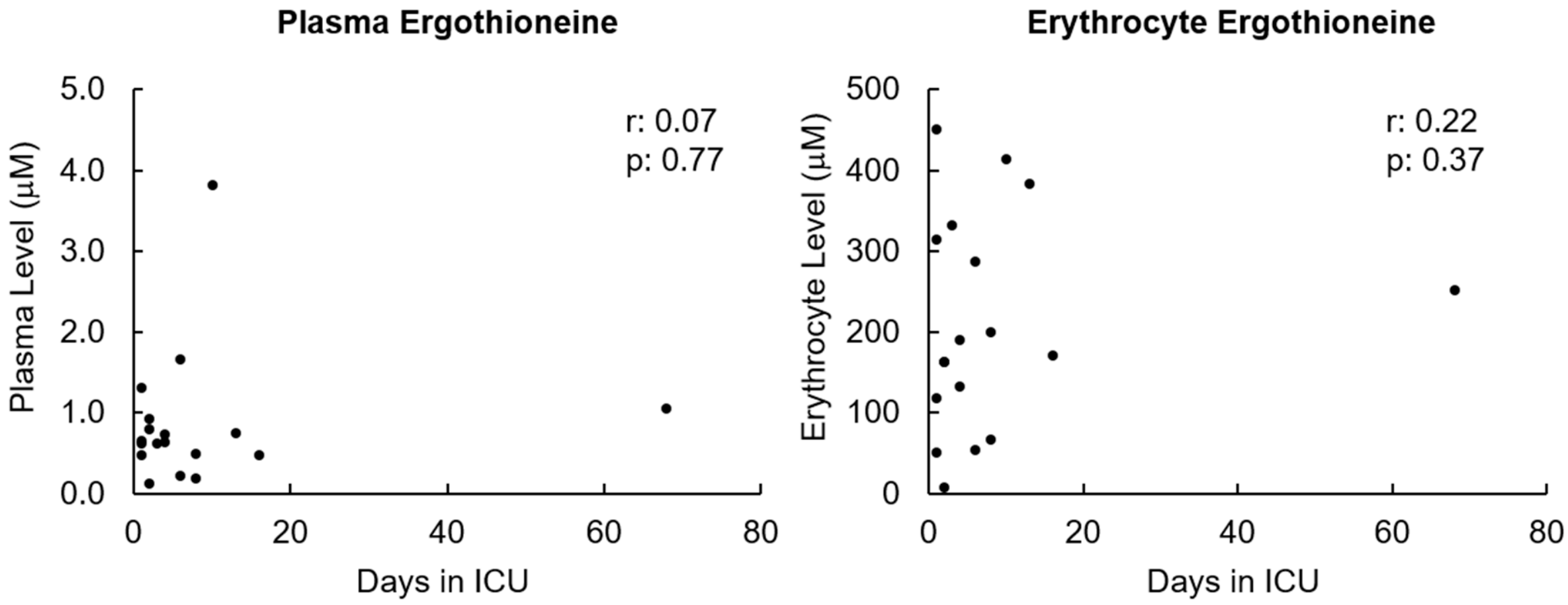

There was no association between ergothioneine levels (left: erythrocyte; right: plasma) and length of intensive care unit (ICU) stay of the 18 critically ill subjects who were not receiving continuous renal replacement therapy. Each dot represents a critically ill subject.
